# Quality Characteristics and Storage Stability of Frying Steak Utilizing Wax-Based Korean Pine Seed Oil

**DOI:** 10.3390/foods13071099

**Published:** 2024-04-03

**Authors:** Peng Wang, Jingyi Wang, Yue Fan, Na Zhang, Qingqi Guo

**Affiliations:** 1 College of Life Science, Northeast Forestry University, Harbin 150040, China; wangpeng981101@163.com (P.W.); fy1449945467@163.com (Y.F.); 2 College of Food Engineering, Harbin University of Commerce, Harbin 150028, China

**Keywords:** Korean pine seed oil, natural wax, butter, fried steak, physical properties, volatile flavor compounds, storage stability

## Abstract

To investigate the disparities in product quality and storage stability between wax-based Korean pine seed oil gel and butter when used for frying steak, a comparative analysis was conducted on cooking loss, color, texture characteristics, sensory evaluation, and volatile flavor substances using headspace solid phase microextraction combined with GM-MS. Furthermore, the storage stability was assessed. The findings revealed that the cooking loss rate of steaks significantly increased with doneness, with butter steak exhibiting a significantly higher loss rate compared to the three oil gel steaks. Hardness, chewiness, and adhesiveness greatly increased as doneness progressed; however, cohesiveness, elasticity, and resilience showed minimal variation. The L* value and b* value of steaks initially increased before stabilizing with increasing doneness levels while the a* value first rose before gradually declining. Medium rare steak received the highest sensory score among all categories tested and 69 volatile flavor compounds were detected. Multivariate data analysis indicated similarities in volatile compounds between butter steak and BW (wax-based Korean pine seed oil gel) steak groups. Additionally, during storage at 4 °C temperature conditions pH level retention water content TVB-N (total volatile basic nitrogen), TBARS (thiobarbituric acid reactive substances) were evaluated to determine advantages or disadvantages within each group: Beeswax (BW) > Carnauba wax (CW) > Rice bran wax (RBW) > butter based on these parameters’ values. It can be concluded that utilizing wax-based Korean pine seed oil gel for frying steaks not only effectively retains significant amounts of unsaturated fatty acids but also preserves steak quality while extending shelf life—a healthier cooking method resulting in reduced oil absorption.

## 1. Introduction

Korean pine (*Pinus koraiensis*) is a prevalent tree species in northeastern China, renowned for its oil-rich seeds. As one of China’s traditional edible oils, Korean pine seed oil (KPSO) boasts high levels of unsaturated fatty acids, vitamin E, beta-sitosterol (a plant sterol), and other potent antioxidants [1]. With a smoke point typically exceeding 180 °C (356 °F), KPSO exhibits remarkable stability during high temperature cooking methods such as stir-frying and deep-frying, minimizing the risk of smoke generation and scorching [2]. By incorporating natural wax into KPSO to form an oil gel, not only can the thermal stability of KPSO be further enhanced but also additional nutritional benefits can be provided [3]. Previous studies have demonstrated that oil gels prepared by blending waxes with vegetable oils offer a viable alternative to animal fat in meat products, including hamburgers and sausages [4,5,6]. Wax-based oil gels can also serve as frying media for product coating. For instance, augmenting the carnauba wax concentration in sunflower oil leads to a reduction in pH value, thereby enhancing product quality [7]. Moreover, edible coatings comprising wax-based rapeseed oil gels exhibit no alterations in water activity and pH levels compared to the original sample [8].

Steak is a high-protein, nutrient dense food that is abundant in essential amino acids required by the human body [9]. Additionally, steak is rich in various B-group vitamins (such as vitamin B_12_, B_6_, niacin) and minerals (including iron, zinc, selenium) [10]. It offers a rich taste and distinctive flavor with versatile cooking methods such as frying, roasting, braising, and boiling [11]. The palatability of meat plays a crucial role in consumer choice [11]. Fried steak provides an enjoyable texture and taste experience [12], but it also has certain drawbacks to consider. High temperature frying can lead to nutrient loss in steak due to the rapid and irreversible processes involved [13]. This may result in overcooked or unevenly cooked steaks that are dry. Furthermore, frying promotes significant chemical and physical changes in the meat. Moreover, prolonged storage time can induce protein and fat oxidation leading to decreased quality and nutrient loss. Therefore, research focusing on enhancing meat quality and extending its shelf life has garnered considerable attention [13]. In Western cuisine, to ensure the tenderness of steak and meet consumer demands, it is typically categorized as rare, medium rare, medium, medium well, or well done [14]. Recent research on steak both domestically and internationally has focused on tenderization [15,16], modified atmosphere packaging [17], and cooking methods [13]. Regarding flavor in steak, studies have concentrated on the impact of surface temperature on volatile flavor compounds in steak [18], the formation of flavor precursors [19], and the role of fat content [20] as well as baking temperature’s effect on volatile flavors and texture [21]. Despite this extensive research, little attention has been given to using oil gels for steaks.

The unique composition of the gel oil enables the formation of a protective oil layer, effectively reducing direct contact between food and high-temperature oil [8]. This not only minimizes nutrient loss during frying and oil absorption but also enhances the tenderness and flavor profile of the food [7]. Furthermore, it imparts a distinctive sensory experience in terms of both taste and texture. KPSO has a nutty taste and light texture, which may provide a unique flavor and texture experience for the steak. Therefore, the objective of this study was to assess the physicochemical properties of naked eye steak fried using KPSO gel. By comparing it with butter-fried steak, various physicochemical properties such as cooking loss, color, texture properties, etc., were evaluated. Additionally, changes in volatile flavor compounds and the sensory quality of the steak during frying were analyzed using headspace solid-phase microextraction gas chromatography-mass spectrometry. Furthermore, storage stability analysis was conducted to provide a reference for quality control of vegetable oil gel fried steaks.

## 2. Materials and Methods

### 2.1. Materials

Frozen naked eye steak (Angus beef, original cut, thickness 2.5 cm) originated from Australia and was provided by Zhiniu Trading Company (Shijiazhuang, China). The organic butter used in the experiment was sourced from J.F.Valt Holding B.V., Netherlands. For the preparation of KPSO gel, three types of waxes, namely Beeswax (BW), Rice bran wax (RBW), and Carnauba wax (CW), were utilized as gelling agents with a weight ratio of 5 g KPSO per group. The addition amount was set at 7 wt%, and the stirring heating process was conducted for 25 min at a temperature of 90 °C [22]. All other chemical reagents utilized in this study were of analytical purity.

### 2.2. Fried Steak

#### 2.2.1. Steak Making Process

Pretreatment: Thaw the steak from a frozen or refrigerated state and allow it to reach room temperature. Firstly, employ a knife to eliminate cartilage, connective tissue, and excessive surface fat. Subsequently, evenly apply 1.8% salt on both sides of the steak and roll it for marination purposes for a duration of 2 h to ensure deep penetration. Finally, slice the marinated steak into 1cm thick pieces. Frying conditions: Heat a pan with butter/KPSO gel at a moderately high temperature (150–170 °C) for frying; transfer the steaks onto a cutting board to slightly cool down to redistribute juices and maintain meat tenderness.

#### 2.2.2. Cooking Method

Three types of KPSO gels, containing 7 wt% BW, RBW, and CW respectively, were used to cook the treated steak on a preheated electric baking pan (F1247 type Zhejiang Donghekang Electrical Appliances Co. Ltd., Yongkang, Zhejiang, China) at 150–170 °C for 480 s after adding 5 g of butter/wax based KPSO gel for spreading and frying. Two thermocouple thermometers (T-105 center thermometer, Shenzhen Tuoerwei Electronic Technology Co. Ltd., Shenzhen, China) were inserted into the center of each steak to measure the center temperatures of five steaks with different degrees of doneness: 50 °C (Rare), 59 °C (Medium Rare), 67 °C (Medium), 74 °C (Medium Well), and 81 °C (Well Done) [23]. The sensory properties were evaluated separately.

### 2.3. Determination of Food Quality

#### 2.3.1. Loss of Cooking

The method described by Agbeniga et al. [10]. was employed with minor adaptations. The surface moisture of the steak was removed using oil absorbing paper, followed by weighing it (*W*_1_). Subsequently, the steak was fried until reaching the desired frying temperature, then cooled to room temperature before draining excess oil and water using oil absorbing paper and re-weighing it (*W*_2_). The cooking loss rate of steaks at various degrees of doneness can be calculated using the following formula (1).

(1) $$Loss\;of\;cooking=\frac{W_{1}-W_{2}}{W_{1}}\times 100\%$$

#### 2.3.2. Microstructure

Select the fried steak with desirable physical properties and employ stereomicroscopes to visually examine the surface oil changes, including the size, shape, and arrangement of the gel cover. Gain a comprehensive understanding of the internal organization and texture of the steak using lenses (2.0×, 3.0×, 5.0×), focusing on crucial features such as texture, muscle fiber arrangement, and fat distribution.

#### 2.3.3. Color Determination

According to the method proposed by Wang et al. [24], the color of the steak was determined using a CR-400 colorimeter (YS3010, Shenzhen Sanenshi Technology Co., Ltd., Shenzhen, China), which was properly calibrated before usage. The colorimeter was positioned vertically on the transverse section surface of the steaks to measure their L*, a*, and b* values. The average value obtained from six measurements was considered as the representative color difference value for each steak.

#### 2.3.4. Texture Properties

The texture analysis method described by Ma et al. [25]. was slightly modified in this study. Steaks of varying degrees of doneness were cut into 1 cm × 1 cm × 1 cm small pieces along the direction of muscle fibers. Texture profiles were analyzed using a P50 probe in “secondary compression” mode on a TA.XT Express Texture Analyzer (StableMicro Systems, London, UK). The following measurement parameters were used: compression ratio of 75%, measurement speed of 1 mm/s, in-test and post-test speeds of 6 mm/s, trigger force set at 5 g, and an interval time of 5 s. Six indices including hardness, elasticity, cohesion, adhesion, chewiness, and resilience were analyzed. The experiment consisted of three samples with varying degrees of doneness, each repeated six times.

#### 2.3.5. Sensory Quality

The sensory evaluation method employed in this study was the sensory overview examination, as outlined in GB/T 22210-2008 “Specification for Sensory Evaluation of Meat and Meat Products” [26]. Thirty-five eligible participants, who were both in good health and non-smokers, were selected as potential evaluators from the student body and faculty members affiliated with the Department of Food Science at Northeast Forestry University (Harbin, China). Ten trained group members (5 men and 5 women, aged between 23 and 57) were selected by using triangulation tests who had passed a course of sensory evaluation. Before aroma evaluation, all evaluators underwent rigorous training sessions on standard fried steak characteristics and sensory evaluation requirements (including definition of quality attributes and scoring methods) for more than one hour every two days over one week to ensure familiarity with steak descriptive terms [12].

Steaks of varying degrees of doneness were fried in different oil samples and cut into 1 cm × 3 cm × 1 cm (thickness) pieces. The samples were stored in an incubator to maintain a temperature of 50 °C and randomly assigned to group members using a cross-over design. Sensory analyses were conducted at room temperature during daylight hours, with separate compartments for each evaluation [27]. Group members first assessed aroma preferences using a predefined assessment protocol established during group training. They were instructed to evaluate the samples based on their color and aroma observations. Before evaluating the next sample, evaluators rinsed their mouths with mineral water to minimize any influence from previous samples, and communication between evaluators was prohibited. Participants then tasted one sample cube and rated its overall impression, taste preference, and texture preference [28]. Subsequently, they tasted a second piece and rated tenderness, juiciness, and off-flavor intensity using a 9-point scale (1 = very weak; 9 = very strong). The left end of the scale was labeled as “very weak”, while the right end was labeled as “very strong” for off-flavor intensity “very juicy” for juiciness, or “very soft” for tenderness ratings. Each session consisted of three sessions within one week where a total of 200 samples across four types with five maturity levels were evaluated.

#### 2.3.6. Determination of Volatile Flavor Substances

According to the results of sensory evaluation, 3 g of sevoflurane meat foam sample was transferred into a 10 mL sample bottle with a TEflon rubber pad. Subsequently, 200 μL 2-methyl-3-heptanone was added. The solid phase microextraction handle needle was then inserted into the sealed sample bottle and equilibrated at 60 °C for 5 min. Afterward, the extraction fiber head was extended and allowed to extract for 55 min using an extraction head (57328-U, Supelco, Bellefonte, PA, USA). Following this step, the extraction head was retracted and the needle swiftly inserted into the GC injection port before desorbing at 250 °C for 3 min. Prior to sampling, it is essential to activate the extraction head in the GC injection port for 5 min [29]. Gas chromatography was performed using an Agilent 6890-5973N GC-MS instrument (Agilent Technologies, Santa Clara, CA, USA). The chromatographic column used was an Agilent innowax column (30 mm × 0.25 mm, 0.25 μm i.d.). The injection port temperature was set at 250 °C with a no split injection method employed. High purity helium served as the carrier gas at a flow rate of 1.0 mL/min. A temperature program was applied, starting from an initial temperature of 40 °C and maintained for 3 min, followed by an increase to 200 °C at a rate of 5 °C/min and further increased to 230 °C at a rate of 10 °C/min, which was then maintained for another 3 min. The total measuring time lasted for approximately 41 min. For mass spectrometry analysis, an electron impact source (EI source) with an electron energy of 70 eV was utilized as the ion source while maintaining the transmission line temperature at 250 °C and ion source temperature at 230 °C. Additionally, the four stage rod mass analyzer operated under a temperature setting of 150 °C, employing scan mode with a mass scanning range between 50~500 amu. Qualitative analysis was conducted using mass spectrometry and retention index. The mass spectrum was determined through computer retrieval and comparison with the NIST02 databases (National Institute of Standards and Technology, Gaithersburg), which was configured with the instrument. Kovats retention indices (RI) of C_5_~C_25_ n-alkanes were calculated by chromatographic scanning under the same chromatographic conditions as the sample determination, followed by comparison with corresponding literature values. RI was calculated according to the following formula (2). Where: *R_t_*_(*i*)_, *R_t_*_(*n*)_, and *R_t_*_(*n*+1)_ are the retention time of the unknown substance to be measured, and the n-alkanes containing n and *n* + 1 carbon atoms, respectively.

(2) $$RI=100\times \left[\frac{R_{t\left(i\right)}-R_{t(n)}}{R_{t\left(n+1\right)}-R_{t(n)}}+n\right]$$

Semi-quantitative analysis: With 2-methyl-3-heptanone as the internal standard, the volatile substance content was calculated according to Equation (3). Where: *ρ_i_* is the mass concentration of the substance to be measured, μg/kg; *A_i_* is the peak area of volatile substances; *A*_0_ was the peak area of 2-methyl-3-heptanone. *ρ*_0_ is the mass concentration of the internal standard solution, μg/kg.

(3) $$\rho_{i}=\frac{A_{i}}{A_{0}}\times \rho_{0}$$

### 2.4. Storage Stability Index

#### 2.4.1. pH

Referring to the national standard GB 5009.237-2016 “Determination of pH value of food for food safety” [30], the following modifications were made. At each time point during storage, four types of medium well steak samples (5 g) were crushed and homogenized with 50 mL of a potassium chloride solution (0.1 mol/L) at a speed of 10 kr/min for 30 s. After allowing it to stand for 5 min, the mixture was filtered and the pH value was measured using a pH meter. Each sample was replicated three times, and the average value was calculated.

#### 2.4.2. Purge Loss

The weight of samples before storage (*m*_1_) and after the sampling date, steak samples were extracted and weighed after removing excess surface water (*m*_2_), enabling calculation of the storage loss rate (4) [31].

(4) $$Purge\;loss\left(\%\right)=\left(\frac{\left(m_{1}-m_{2}\right)}{m_{1}}\right)\times 100\%$$

#### 2.4.3. TVB-N

The determination method was conducted following the protocol outlined in GB 5009.228-2016, titled “Determination of total volatile salt base nitrogen in Food” [32]. Ten grams of samples were finely minced and transferred into a conical flask, followed by the addition of 50 mL of distilled water. After shaking for 15 min, the mixture was left at room temperature for 20 min. Subsequently, the filtrate was collected and subjected to filtration before determining the TVB-N value of beef samples using a semi-trace nitrogen distillation device.

#### 2.4.4. TBARS

The determination of thiobarbituric acid reactants (TBARS) was conducted following the method described by Etemadian et al. [33]. Briefly, a 1.0 g sample was weighed, ground, and homogenized. Subsequently, 5 mL of 7.5% trichloroacetic acid was added to the sample and stirred for 10 min to ensure even dispersion. After two rounds of filtration, 1 mL of 0.375% 2-thiobarbituric acid was introduced into the supernatant and mixed thoroughly before being subjected to a boiling water bath for 30 min. Following this step, the mixture was allowed to cool at room temperature and then centrifuged at a speed of 6000 r/min for 20 min. Finally, the absorbance of the resulting supernatant was measured at a wavelength of 532 nm using spectrophotometry. Each experimental group performed three parallel runs.

### 2.5. Statistical Analysis

Each experiment was conducted three times and expressed as mean ± standard deviation values. Statistical analysis was performed using the Origin software (2018, OriginLab, Northampton, MA, USA) package with one-way analysis of variance (ANOVA) followed by the Woller–Duncan post hoc test (*p* < 0.05), utilizing the SPSS software (SPSS26; IBM SPSS Statistics, Chicago, IL, USA) statistical program.

## 3. Results

### 3.1. Changes in Cooking Losses

**Figure 1:**
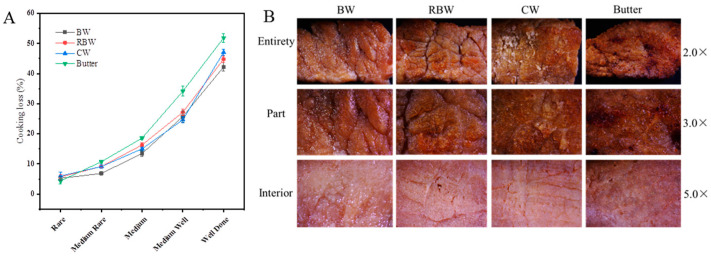
Cooking loss rates of different waxy based KPSO gels versus Butter steak (**A**); Schematic of medium well microstructure (**B**).

The variation in cooking loss rates among different KPSO gels and Butter steak is depicted in Figure 1A. The rare steak exhibited the lowest cooking loss rate, with values of 5.22% for BW steak, 5.68% for RBW steak, 6.01% for CW steak, and 4.33% for Butter steak. Conversely, the well-done steaks displayed the highest cooking loss rates: 42.21% for BW steaks, 44.82% for RBW steaks, 47.09% for CW steaks, and 51.86% for Butter steaks. The cooking loss rate of the steak with Butter is observed to be higher compared to that of the steak with oil gel group. This disparity can be attributed to the presence of a wax-based coating in the oil gel group, which exhibits superior water retention properties. Consequently, this enhanced water retention capability safeguards the steak from gravy loss and subsequently reduces overall losses. When considering medium well and well done states, it was observed that the cooking loss rate was significantly higher in the Butter group compared to the gel group (*p* < 0.05). This can be attributed to an increase in meat juice discharge within beef during frying [18]. The reduction in released meat juice during chewing leads to decreased chewiness and tenderness of the steak; thus a higher degree of doneness results in greater center temperature elevation leading to increased water loss within beef affecting its quality as reflected by elevated cooking loss rates. Similar findings have been reported regarding pork studies [34].

The microstructure changes of medium well steaks are illustrated in Figure 1B. The muscle fibers of the oil gel treated steaks exhibited a more compact arrangement compared to those of the Butter treated steaks. Moreover, a majority of the perimysial fibers appeared curved and irregularly arranged, particularly with tightly enveloped muscle fiber bundles and significantly reduced enimysial space [20]. Conversely, there was a substantial gap between the perimysium in Butter treated steaks, leading to inferior chewiness. Notably, distinct variations were observed in the organization of muscle fibers among beef samples subjected to different oil frying treatments. Muscle fibers in RBW and CW displayed swelling effects along with weakened connective tissue and water loss, resulting in increased inter-fiber spacing [3]. Consequently, it can be inferred that steaks cooked using an oil gel method exhibit enhanced tenderness.

### 3.2. TPA

**Figure 2:**
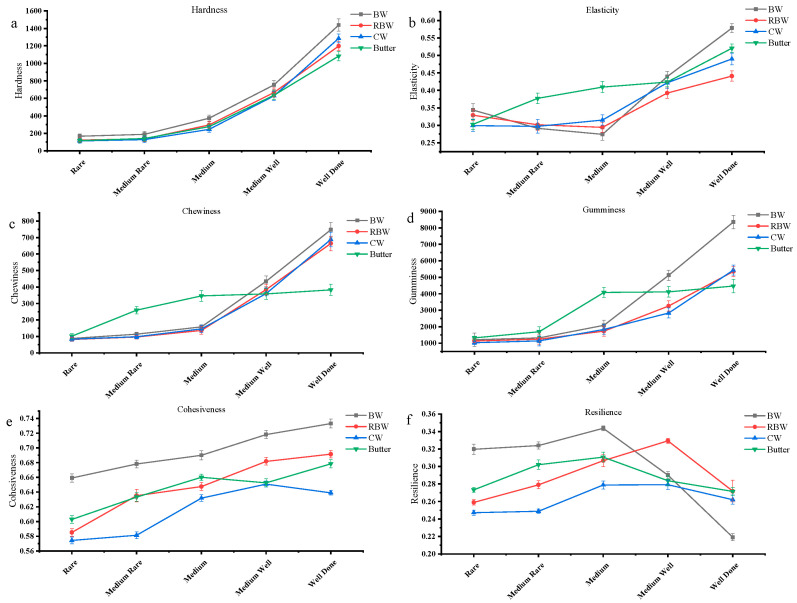
KPSO gel and Butter steak texture index diagram, Hardness (**a**); Elasticity (**b**); Chewiness (**c**); Gumminess (**d**); Cohesiveness (**e**); Resilience (**f**).

Texture profile analysis (TPA) is a method that mimics the mastication process in the human oral cavity, serving as a direct indicator to assess the textural attributes of food [35]. Tenderness plays a crucial role in evaluating steak quality and TPA enables an objective evaluation of its tenderness [36]. Figure 2 demonstrates significant variations in certain texture properties between KPSO gel and Butter steak (*p* < 0.05). Hardness, chewiness, and stickiness exhibit notable differences based on doneness levels with rare steak displaying the lowest values. Except for rare and medium rare, the hardness, chewiness, and stickiness of fried steaks significantly increased with higher degrees of cooking (*p* < 0.05). However, the hardness of BW steaks in the gel group changed greatly when cooked to well done. The chewiness of BW steaks increased as they ripened. The chewiness and resilience of Butter steaks (Figure 2c,e) differed significantly among rare, medium rare, and medium matured steaks (*p* < 0.05). Only hardness showed a significant difference between medium well and well done (*p* < 0.05). Figure 2a–d indicates no significant difference between rare Butter steaks and gel steamed ones; however, when fried to medium well or well done levels, texture characteristics were significantly higher for gel steamed than for Butter steamed ones (*p* < 0.05), due to surface myofibril contraction caused by the gel group that led to the rapid formation of a shell membrane. The rapid formation of a shell film during the initial stage of steak frying using oil gel [37] effectively mitigated water loss in subsequent stages and also directly influenced cohesion differences (Figure 2e). Oil gel enhanced the water retention capacity of the steak while promoting minimal internal hardening. Furthermore, it was observed that there were negligible distinctions between Butter steaks and gel steaks cooked to medium well doneness.

### 3.3. Color and Luster Change

The color of steak is influenced by its degree of cooking, as indicated in Table 1. As the degree of cooking increases, the L* and b* values initially increase and then stabilize [38]. However, when the steak is cooked to medium doneness, there are no significant changes observed in the L* and b* values (*p* > 0.05). This can be attributed to the gradual formation of a surface film on the steak during frying which prevents further color changes. Additionally, leakage of steak juice during cooking enhances brightness by increasing the L* value. With an increase in the internal temperature of the steak, fat oxidation intensifies and surface film becomes more coked; consequently, there are no significant changes observed in both L* and b*. On the other hand, a* initially increases with increasing degree of cooking due to the decoking of surface membrane early on while heating promotes myoglobin oxidation leading to an increase in a*. In later stages, however, intensified coking of the surface membrane results in oxidation towards brown high iron myoglobin causing darkening of steak color [5], resulting in a significant decrease in a* (*p* > 0.05). Furthermore, according to Table 1, the L* values for Butter steaks ranging from rare to well done were higher compared to gel steaks whereas gel steaks exhibited higher a* values at rare stage than Butter steaks did. When fried between medium rare~medium well except for BW steaks, a* values for Butter steaks were lower than those for RBW and CW steaks. Varying degrees of change were observed for both Butter steaks and gel steaks regarding their b* value as maturity progressed.

### 3.4. Sensory Assessment

According to the results presented in Table 2, an increase in steak doneness was observed to be associated with a gradual improvement in color, flavor, chewiness, and overall impression of cooked meat. However, tenderness and juiciness showed a gradual decrease [24]. These findings suggest that as doneness increases, longer heating times promote the development of desirable attributes such as color, taste, and aroma; however, they also contribute to a gradual aging effect on the taste of the steak. The higher overall impression with increasing doneness (in contrast to decreasing tenderness) can be attributed mainly to the preference of sensory evaluation panel members for steaks with higher levels of doneness. This aligns with previous research by Lang et al. [12], who reported significantly higher scores for flavor and overall impression in steaks cooked at higher endpoint central temperatures (72~100 °C) compared to those cooked at lower endpoint central temperatures (45 and 60 °C) (*p* < 0.05). Although rare and medium rare steaks exhibit relatively low hardness and tenderness values, their degree of cooking is insufficient to meet the consumption preferences of most Chinese consumers [27]. The medium steak is deemed acceptable by certain individuals. The sensory indexes indicate that the medium well steak has the highest score, with appropriate levels of hardness and tenderness, making it easily accepted by consumers. Additionally, as the internal temperature of the steak increases, the Maillard reaction occurs, resulting in a release of aroma [25]. During this process, the steak retains a certain level of moisture content and becomes juicier. Comparing steaks cooked to the same doneness level, those coated with gel exhibited significantly higher sensory scores than Butter coated steaks (*p* < 0.05), particularly for well done gel coated steaks compared to Butter coated ones. These findings suggest that wax-based coatings can enhance the color, flavor, chewiness, and overall impression of cooked meat. The overall sensory evaluations indicated that steaks coated with butter and gel achieved the highest scores when cooked to a medium well level.

### 3.5. Volatile Flavor Compounds

**Figure 3:**
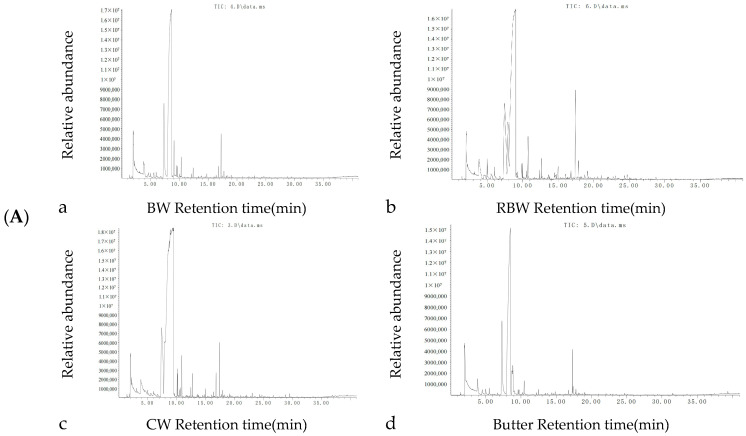

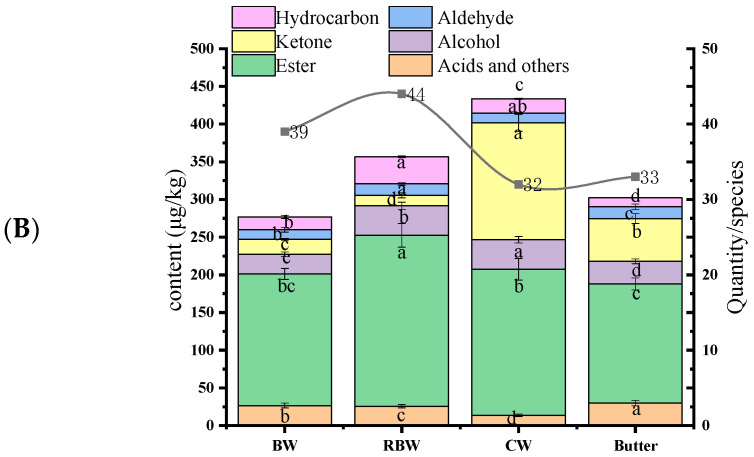
Total ion current diagram of volatile components of steak by headspace solid phase microextraction (**A**); Content and quantity of volatile substances in steak (**B**) (Note: different letters indicate significant differences within groups (*p* < 0.05)).

The total ion current map of volatile components in KPSO gel and Butter fried steak (Figure 3A) demonstrated a consistent chromatographic peak response for each volatile component, exhibiting comprehensive chromatographic information, effective separation, moderate retention time, and concentration within 30 min [39]. Based on the peak time and area of each volatile component, the relative content of individual constituents was calculated.

A total of 69 volatile flavor compounds were detected from medium well steaks, including 18 hydrocarbons, 10 alcohols, 9 aldehydes, 9 ketones, 6 acids, 11 esters, and 6 others. Figure 3B illustrates that the concentration of volatile flavor compounds in BW was the lowest at (276.85 ± 20.10) μg/kg compared to RBW at (356.55 ± 28.97) μg/kg and CW at (433.60 ± 33.10) μg/kg, respectively; Butter exhibited a content of (302.22 ± 24.89) μg/kg which was intermediate among these samples analyzed for comparison purposes. The content of BW samples demonstrated lower levels than those observed in RBW samples while RBW exhibited the highest concentration of volatile flavor substances among all tested samples analyzed herein. The number of volatile flavor compounds identified in steaks was as follows: BW (39), RBW (44), CW (32), and Butter (33). Notably, CW samples displayed a significantly reduced diversity of volatile flavor compounds compared to Butter samples with slightly lower concentrations potentially attributed to thermal degradation or formation of other compounds during further heating processes as previously reported by Wall et al. [20]. This phenomenon may also be associated with extensive volatilization caused by prolonged heating conditions as suggested by Mao et al. [39]. Further investigations are warranted to elucidate specific underlying mechanisms.

According to Table 3, significant differences were observed in the volatile flavor substance content and quantity between KPSO gel and Butter fried steaks. In BW, RBW, and CW samples, the volatile flavors primarily consisted of alene and alkane compounds [35]. The detected hydrocarbons (Table 3-A) exhibited significant variations in both quantity and content. These differences could be attributed to prior fatty acid degradation or amino acid oxidation processes [40]. Additionally, Tetradecane, 2-di(propan-2-yl)phosphorylpropane, and trans-Caryophyllene were co-detected as hydrocarbons. It is worth noting that certain other hydrocarbons may serve as precursors for heterocyclic compounds that can potentially influence the overall flavor profile of steak [35]. In terms of aldehydes (Table 3-B), four aldehydes were simultaneously detected in all four samples, exhibiting significantly different contents despite a substantial degree of overlap. The shared aldehydes included Nonanal, (2E)-2-Octenal, (E)-2-Decenal, and (2E,4E) -deca-2,4-dienal. Notably, Nonanal is an oxidation product derived from oleic acid [41], imparting a fried and fatty flavor; whereas (2E,4E) -deca-2,4-dienal contributes to a greasy and flesh-like aroma. These aldehydes primarily originate from fat oxidation processes as well as the degradation and Strecker reaction of amino acids [27,42], playing a pivotal role in the development of steak flavor. As for the ketones (Table 3-C), two common ketones were identified: 2,2′,7-trimethyl-3′,5′-octanedione and 4-octanone. While most ketones possess higher thresholds and contribute less to the overall flavor profile, certain ketones serve as crucial intermediates in heterocycle formation processes [41] that contribute to meat flavor development.

Regarding the alcohol substances (Table 3-D), significant variations were observed in the compositions of the four samples, with 1-Octen-3-ol, 2-(2-ethoxyethoxy)-ethanol, and 3-Octanol being identified as the predominant compounds. These alcohols play a crucial role in shaping the overall flavor profile of steak [42]. For instance, 1-Octen-3-ol, derived from linoleic acid hydroperoxide degradation, contributes to a distinct mushroom aroma [43] and significantly influences the flavor characteristics of steak. The interaction between alcohols and free fatty acids during steak lipization produces esters (Table 3-E), which contribute to the fatty, waxy, and floral aroma of steaks [23]. There are significant differences in ester profiles among the four samples, with their contribution to the overall aroma being less pronounced compared to that of aldehydes. However, they do enhance the overall aroma complexity of the steak [44]. Furthermore, variations in types and contents of acids (Table 3-F) among the four samples primarily result from fat degradation and oxidation processes [35,40]. While some acids may possess unpleasant odors, other heterocyclic compounds exhibit meaty characteristics and play a crucial role in shaping the overall aroma profile [25,44]. Overall, these volatile flavor substances not only provide a rich layer of complexity to the flavor of steak but also reflect the intricate diversity arising from various chemical reactions occurring during cooking.

**Table 3:** Comparison of volatile flavor substances between KPSO gel and Butter steak.

Category	Peak#	Compound	Molecular Formula	CAS	KI	R	Content/(μg·kg^−1^)			
							BW	RBW	CW	Butter
Hydrocarbon (A)	A1	n-Hexane	C_6_H_14_	110-54-3	618	m,s,k	1.67 ± 0.03 ^a^	1.54 ± 0.05 ^bc^	1.54 ± 0.01 ^c^	ND
	A2	2-ethyl-3-methyl-oxetane	C_6_H_12_O	53778-62-4	724	m	ND	1.66 ± 0.05 ^b^	2.16 ± 0.18 ^a^	ND
	A3	Cyclohexane,(1,1-dimethylethyl)-	C_10_H_20_	3178-22-1	762	m	ND	2.81 ± 0.09 ^a^	2.70 ± 0.08 ^b^	ND
	A4	octylcyclopropane	C_11_H_22_	1472-09-9	732	m	ND	ND	2.19 ± 0.04	ND
	A5	1,1,2-trimethylcyclopentane	C_8_H_16_	4259-00-1	1148	m	ND	4.67 ± 0.16	ND	ND
	A6	Tetradecane	C_14_H_30_	629-59-4	1448	m,s,k	1.31 ± 0.17^d^	3.19 ± 0.21 ^a^	1.90 ± 0.05 ^c^	2.99 ± 0.05 ^b^
	A7	pentadecane	C_15_H_32_	629-62-9	1512	m,s,k	ND	1.75 ± 0.02	ND	ND
	A8	Hexadecane	C_16_H_34_	544-76-3	1612	m,s,k	ND	2.28 ± 0.09 ^a^	ND	1.72 ± 0.06 ^b^
	A9	1-Iodononane	C_9_H_19_I	4282-42-2	1224	m	ND	1.54 ± 0.06	ND	ND
	A10	Nonylcyclopropane	C_12_H_24_	74663-85-7	862	m	1.61 ± 0.17 ^a^	1.74 ± 0.02 ^b^	ND	ND
	A11	Heptane, 4,4-dimethyl-	C_9_H_20_	1068-19-5	1426	m	2.75 ± 0.47 ^a^	1.74 ± 0.02 ^b^	ND	ND
	A12	2-di(propan-2-yl)phosphorylpropane	C_9_H_21_OP	17513-58-5	1244	m	1.70 ± 0.13 ^c^	1.72 ± 0.13 ^b^	2.17 ± 0.14 ^a^	1.66 ± 0.03 ^d^
	A13	2,4,4,6-tetramethyl-hept-2-ene	C_11_H_22_	103982-58-7	1140	m,k	ND	1.57 ± 0.09 ^b^	3.12 ± 0.18 ^a^	ND
	A14	1-Tetradecene	C_14_H_28_	1120-36-1	1428	m,s	1.33 ± 0.12 ^b^	ND	ND	1.66 ± 0.01 ^a^
	A15	α-Copaene	C_15_H_24_	3856-25-5	1221	m,s,k	ND	ND	ND	1.88 ± 0.12
	A16	(+)-Limonene	C_10_H_16_	5989-27-5	1018	m,s,k	ND	3.27 ± 0.07 ^a^	1.51 ± 0.06 ^b^	ND
	A17	(1Z,3Z)-1,4-dimethoxy-1,3-butadiene	C_6_H_10_O_2_	83650-30-0	1140	m	1.86 ± 0.14 ^b^	4.49 ± 0.11 ^a^	ND	ND
	A18	trans-Caryophyllene	C_15_H_24_	87-44-5	1494	m,s,k	4.76 ± 1.11 ^a^	1.88 ± 0.05 ^c^	1.92 ± 0.14 ^b^	1.80 ± 0.08 ^d^
Aldehydes (B)	B1	1-hexadecanal	C_16_H_32_O	629-80-1	1728	m,k	ND	ND	1.67 ± 0.14	ND
	B2	Nonanal	C_9_H_18_O	124-19-6	1104	m,s,k	4.30 ± 1.69 ^b^	3.53 ± 0.09 ^c^	1.46 ± 0.03 ^d^	5.07 ± 5.35 ^a^
	B3	(2E)-2-Octenal	C_8_H_14_O_2_	2548-87-0	1013	m,s,k	1.93 ± 0.87 ^a^	1.87 ± 0.04 ^b^	1.73 ± 0.11 ^c^	1.86 ± 0.14 ^b^
	B4	(E)-2-Decenal	C_10_H_18_O	3913-81-3	1242	m	1.70 ± 0.06 ^bc^	1.59 ± 0.23 ^c^	1.76 ± 0.14 ^a^	1.71 ± 0.16 ^b^
	B5	Benzaldehyde	C_7_H_6_O	100-52-7	982	m,s,k	ND	2.25 ± 0.15 ^a^	2.14 ± 0.18 ^b^	ND
	B6	Tridecanal	C_13_H_26_O	10486-19-8	1502	m,s,k	ND	1.99 ± 0.14 ^b^	ND	3.63 ± 2.71 ^a^
	B7	2-Isopropylbutanal	C_7_H_14_O	26254-92-2	1095	m	1.79 ± 0.13 ^b^	ND	2.03 ± 0.13 ^a^	ND
	B8	1-Nonana	C_9_H_18_O	124-19-6	1104	m	1.36 ± 0.06 ^b^	ND	ND	1.76 ± 0.16 ^a^
	B9	(2E,4E)-Deca-2,4-dienal	C_10_H_16_O	25152-84-5	1195	m	1.57 ± 0.36 ^c^	3.90 ± 0.48 ^a^	1.82 ± 0.11 ^b^	1.51 ± 0.08 ^d^
Ketone (C)	C1	Butyl 2-methyl-1-propenyl ketone	C_9_H_16_O	19860-71-0	847	m	1.43 ± 0.02 ^b^	ND	1.71 ± 0.14 ^a^	ND
	C2	Isobutyrylacetone	C_6_H_10_O_2_	7307-03-1	728	m	1.43 ± 0.06 ^c^	ND	146.73 ± 11.57 ^a^	39.61 ± 7.48 ^b^
	C3	1-(6-methyl-3,4-dihydro-2H-pyran-2-yl)propan-1-one	C_9_H_14_O_2_	62255-24-7	764	m	2.88 ± 0.18 ^b^	ND	ND	5.65 ± 1.21 ^a^
	C4	1-(2-Hydroxy-6-methoxyphenyl)ethanone	C_9_H_10_O_3_	703-23-1	837	m	ND	2.77 ± 0.19 ^a^	ND	2.64 ± 0.61 ^b^
	C5	2,2,7-trimethyl-3,5-octanedione	C_11_H_20_O_2_	69725-37-7	984	m	6.90 ± 0.78 ^a^	2.02 ± 0.19 ^c^	1.49 ± 0.06 ^d^	2.89 ± 0.35 ^b^
	C6	2,5-Piperazinedione,3-methyl-(9CI)	C_5_H_8_N_2_O_2_	6062-46-0	652	m	ND	3.28 ± 2.49	ND	ND
	C7	3-methyl-Cyclohexanone	C_7_H_12_O	591-24-2	717	m	1.70 ± 0.06 ^a^	ND	ND	1.44 ± 0.12 ^b^
	C8	4-Octanone	C_8_H_16_O	589-63-9	952	m,k	4.22 ± 0.12 ^cd^	5.87 ± 0.54 ^a^	5.12 ± 2.91 ^b^	4.30 ± 0.15 ^c^
	C9	3-Nonen-2-one	C_9_H_16_O	14309-57-0	1188	m	1.34 ± 0.03	ND	ND	ND
Alcohol (D)	D1	4-chloro-2-methylbutan-2-ol	C_5_H_11_ClO	1985-88-2	890	m,k	ND	ND	1.95 ± 0.04	ND
	D2	4-Hydroxy-4-methyl-2-pentanone	C_6_H_12_O_2_	123-42-2	874	m	ND	ND	ND	1.52 ± 0.01
	D3	2-Hexyl-1-decanol	C_16_H_34_O	2425-77-6	1159	m	1.39 ± 0.05 ^b^	1.60 ± 0.06 ^a^	ND	ND
	D4	2-methyl-2-tetradecanol	C_15_H_32_O	27570-83-8	1854	m	ND	1.89 ± 0.11	ND	ND
	D5	2-Dodecen-1-ol	C_12_H_24_O	22104-81-0	1107	m	ND	2.00 ± 0.13	ND	ND
	D6	1-Octen-3-ol	C_8_H_16_O	3391-86-4	969	m,s,k	1.45 ± 0.11 ^d^	2.71 ± 0.04 ^a^	1.92 ± 0.14 ^b^	1.73 ± 0.18 ^c^
	D7	3-Octanol	C_8_H_18_O	589-98-0	1064	m,k	1.75 ± 0.19 ^c^	1.85 ± 0.09 ^a^	1.72 ± 0.06 ^d^	1.80 ± 0.11 ^b^
	D8	4-Isopropoxybutanol	C_7_H_16_O_2_	31600-69-8	928	m	3.35 ± 0.82 ^d^	5.93 ± 0.59 ^a^	3.80 ± 0.17 ^c^	4.38 ± 0.04 ^b^
	D9	2-(2-Ethoxyethoxy)ethanol	C_6_H_14_O_3_	111-90-0	842	m	1.52 ± 0.05 ^d^	2.07 ± 0.61 ^b^	1.81 ± 0.06 ^c^	2.66 ± 0.12 ^a^
	D10	1-Hexanol	C_6_H_14_O	111-27-3	860	m,s,k	16.53 ± 3.89 ^d^	20.16 ± 3.44 ^b^	28.16 ± 6.82 ^a^	18.53 ± 4.06 ^c^
Esters (E)	E1	Octanoic acid, 2-propyl-, methyl ester, (2R)	C_12_H_24_O_2_	946516-84-3	973	m,s,k	1.46 ± 0.08 ^b^	ND	5.34 ± 1.24 ^a^	ND
	E2	Butyl Valerate	C_9_H_18_O_2_	591-68-4	1381	m,s,k	1.66 ± 0.17 ^d^	3.05 ± 1.054 ^b^	2.83 ± 0.01 ^c^	4.29 ± 0.21 ^a^
	E3	heptyl 2,2,2-trifluoroacetate	C_9_H_15_F_3_O_2_	2710-89-6	1164	m	ND	ND	1.52 ± 0.19 ^b^	2.96 ± 0.51 ^a^
	E4	Carbonic acid, butylethyl ester	C_7_H_14_O_3_	30714-78-4	972	m	15.32 ± 2.51	ND	ND	ND
	E5	Pentanoic acid,1-methylethyl ester	C_8_H_16_O_2_	18362-97-5	1481	m	124.1 ± 1.85 ^d^	202.94 ± 14.37 ^a^	157.92 ± 13.47 ^b^	141.17 ± 6.47 ^c^
	E6	Pentanoic acid, propylester	C_8_H_16_O_2_	141-06-0	1481	m	6.43 ± 0.084 ^a^	5.30 ± 0.22 ^c^	5.86 ± 0.23 ^b^	3.88 ± 0.43 ^d^
	E7	Hexyl valerate	C_11_H_22_O_2_	1117-59-5	1780	m	3.51 ± 0.16 ^c^	4.20 ± 0.13 ^a^	3.06 ± 0.13 ^d^	3.75 ± 0.14 ^b^
	E8	2,4-dimethylpentan-3-yl 2-methylpropanoate	C_11_H_22_O_2_	87386-67-2	1914	m	1.51 ± 0.11 ^c^	3.10 ± 0.34 ^a^	1.79 ± 0.14 ^b^	ND
	E9	Pentanoic acid, pentyl ester	C_10_H_20_O_2_	2173-56-0	1814	m	2.65 ± 0.32 ^c^	4.77 ± 0.28 ^a^	3.20 ± 0.32 ^b^	ND
	E10	4-Heptanolide	C_7_H_12_O_2_	105-21-5	1582	m	19.00 ± 3.24 ^a^	ND	13.37 ± 1.05 ^b^	2.09 ± 0.04 ^c^
	E11	cyclobutanecarboxylic acid cyclobutyl ester	C_9_H_14_O_2_	42392-30-3	1282	m	1.68 ± 0.33 ^b^	3.51 ± 0.67 ^a^	ND	ND
Acids and other classes (F)	F1	Pentanoic acid	C_5_H_10_O_2_	109-52-4	1126	m,s,k	ND	ND	2.00 ± 0.25 ^b^	2.68 ± 0.09 ^a^
	F2	Lauric acid	C_12_H_24_O_2_	143-07-7	958	m	ND	ND	1.63 ± 0.23	ND
	F3	Eugenol	C_10_H_12_O_2_	97-53-0	1392	m,s,k	ND	ND	ND	2.11 ± 0.17
	F4	Nonanoic acid	C_9_H_18_O_2_	112-05-0	1272	m	ND	ND	ND	1.82 ± 0.07
	F5	1,2-Benzenedicarboxylic acid	C_16_H_22_O_4_	84-74-2	1482	m	ND	ND	ND	3.11 ± 0.24
	F6	Palmitic acid	C_16_H_32_O_2_	57-10-3	1479	m,s,k	1.28 ± 0.05 ^b^	ND	ND	1.89 ± 0.21 ^a^
	F7	Toluene	C_7_H_8_	108-88-3	786	m	2.83 ± 0.19 ^d^	11.16 ± 1.15 ^a^	4.25 ± 0.38 ^c^	7.60 ± 0.58 ^b^
	F8	n-Valeric anhydride	C_10_H_18_O_3_	2082-59-9	1108	m	ND	2.66 ± 0.08 ^a^	1.66 ± 0.21 ^b^	ND
	F9	2-Amylfuran	C_9_H_14_O	3777-69-3	983	m	1.45 ± 0.11 ^c^	1.96 ± 0.11 ^a^	1.54 ± 0.13 ^b^	1.72 ± 0.12 ^ab^
	F10	Pyridine	C_5_H_5_N	110-86-1	852	m	16.84 ± 4.37 ^a^	4.04 ± 0.56 ^c^	ND	3.59 ± 0.77 ^b^
	F11	Naphthalene	C_10_H_8_	91-20-3	1122	m	1.56 ± 0.13 ^c^	2.92 ± 0.02 ^a^	ND	2.90 ± 0.03 ^b^
	F12	cis-Anethol	C_10_H_12_O	104-46-1	1190	m,s,k	1.39 ± 0.19 ^c^	2.54 ± 0.58 ^ab^	2.16 ± 0.37 ^b^	2.93 ± 0.38 ^a^

Note: ND indicates not detected, values with different lowercase letters in the same column are significantly different (*p* < 0.05). KI: Kovats index calculated for DB-624 capillary column (J&W scientific: 30 m × 0.25 mm id, 1.4 μm film thickness) installed on a gas chromatograph equipped with a mass selective detector; R: Reliability of identification; k: Kovats index in agreement with literature [45]. m: mass spectrum agreed with mass database (NIST02); s: mass spectrum and retention time identical with an authentic standard.

**Figure 4:**
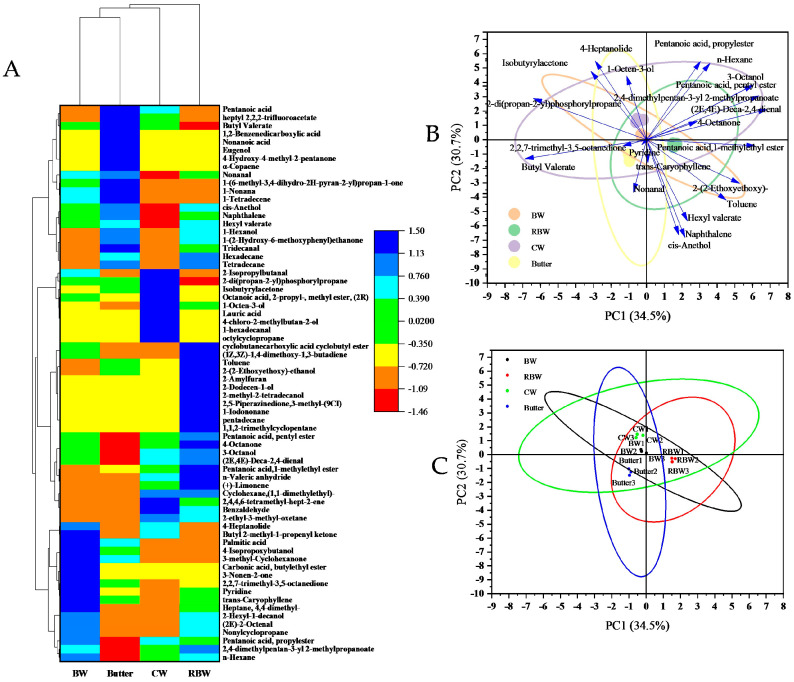
Flavor cluster analysis diagram (**A**); PCA analysis chart (**B**,**C**).

To visually represent the changes in volatile flavor substance content, a heat map analysis was conducted on the volatile flavor substances, as depicted in Figure 4A. The color gradient from blue to red indicates decreasing levels of substance content. A total of sixty-nine substances were detected, including 2-ethyl-3-methyl-oxetane, 1-hexadecanal, Butyl 2-methyl-1-propenyl ketone, 4-chloro-2-methylbutan-2-ol, Octanoic acid, methyl ester (2R), and Pentanoic acid. Among these compounds, twenty-one common flavor compounds were identified suggesting minimal impact on flavor due to oil gel frying. Additionally, forty-eight substances were not commonly detected such as octylcyclopropane, pentadecane Tridecanal, and 2,5-piperazinedione (3-methyl-(9CI)). Furthermore, there are twenty-eight substances exclusively present in KPSO gels but absent in Butter steaks which exhibit fluctuating patterns based on the wax base.

According to Figure 4A, the cluster analysis of the four samples revealed that BW and Butter exhibited close clustering, as indicated by a decrease in Manhattan distance. Generally, the samples were categorized into three groups: BW and Butter, CW and RBW. The volatile flavor substances in oily steak displayed significant differences, potentially attributed to the generation of novel compounds primarily comprising alcohols and ketones resulting from fatty acid oxidation and amino acid degradation. Some examples include Nonylcyclopropane, Heptane, 4,4-dimethyl-, 2-(2-ethoxyethoxy)-, Toluene, Pentanoic acid,1-methylethyl ester. These substances are prone to oxidation or intermolecular reactions [41]. Additionally, principal component analysis (PCA) was employed to discern the disparities in volatile flavor components during the steak frying process. The peak volume of volatile flavor substances served as the data source, and PCA Figure 4B,C was presented to scrutinize the contribution of each flavor compound towards similarities and differences. By examining the loadings of individual flavor compounds (Figure 4B), it was observed that the established PCA model segregated flavors into two principal components, with a contribution rate of 34.50% for principal component 1 and 30.70% for principal component 2, resulting in a cumulative contribution rate of 65.20%. Notably, there were notable variations in terms of flavor compound contributions towards differences along the PC2 direction. Among these compounds, 2 -(2-Ethoxyethoxy)ethanol, Toluene, Hexyl valerate, Naphthalene, and cis-Anethol exhibited significant influence on differentiating RBW from Butter steak [15]. The flavor spectra of RBW and Butter exhibit significant differences, as depicted in Figure 4C. Additionally, the samples of BW, RBW, CW, and Butter are distinctly separated into four different quadrants based on the direction of PC1. Through load chart analysis, it was determined that RBW (2,5-piperazinedione,3-methyl-(9CI)), BW (3-Nonen-2-one), Butter (4-Hydroxy-4-methyl-2-pentanone), and CW (Lauric acid) were the key flavor compounds contributing to these pronounced differences. Notably, the coordinates of the 12 sample points from each group varied significantly within the coordinate system with some overlapping observed among groups. This suggests a certain degree of similarity in steak frying flavors which is consistent with cluster analysis results. In particular, BW and Butter exhibited close distribution patterns along principal component 1 indicating similar flavors while RBW displayed distinct flavor characteristics compared to the other three samples.

### 3.6. Storage Stability

**Figure 5:**
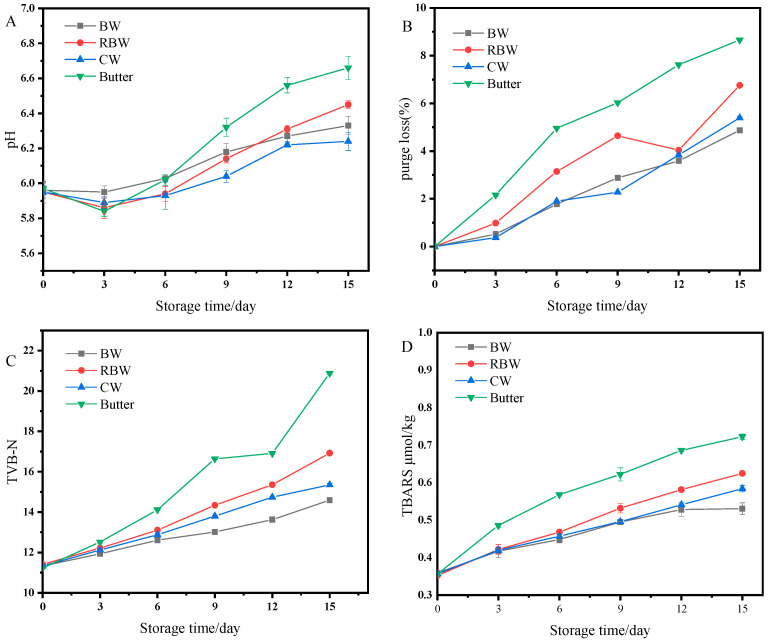
Changes in storage stability indicators, pH (**A**), purge loss (**B**),TVB-N (**C**), TBARS (**D**).

With prolonged storage time, the pH value of steak generally exhibits an upward trend (Figure 5A), attributed to bacterial and enzymatic activity in the meat, leading to protein decomposition into alkaline substances such as ammonia and amine compounds [46]. This increase in pH value is counteracted by the abundant growth and multiplication of microorganisms during later stages of storage, resulting in the production of acidic metabolites that neutralize alkaline substances like amine compounds [47]. Figure 5A also demonstrates distinct variations in pH change rates among different groups of steak meat. The initial pH was recorded as 5.96. From the sixth day onwards, the Butter group exhibited a rapid rise with a pH reaching 6.69 on the twelfth day when it had deteriorated. After fifteen days of storage, the RBW group had a pH value of 6.45 while the CW and BW groups had values of 6.23 and 6.33, respectively. It can be observed that oil gel frying technology effectively controls beef’s pH increase by isolating its surface from the air for gas exchange purposes; this prevents external microbial invasion while freezing temperatures inhibit enzyme activity thereby reducing alkaline substance production [48]. According to Figure 5B, the storage loss rate of fried steaks in the four groups exhibited a significant increase with prolonged storage time. This can be attributed to the denaturation of protein in steak during storage [47], resulting in damage to its spatial structure and consequently leading to an elevated storage loss rate and reduced water retention [40]. After a storage period of 15 days, the order of storage loss rates among the groups was observed as Butter (8.66%) >RBW (6.75%) >CW (5.39%) >BW (4.87%). Notably, the highest storage loss rate was observed in the Butter group, indicating that using butter would increase both the storage loss rate and reduce water retention in beef samples. Conversely, the BW group exhibited significantly lower levels of storage loss, which could effectively minimize surface dryness and internal component diffusion within steaks [49], thereby better preserving water retention.

The increase in total volatile basic nitrogen (TVB-N) is attributed to the deacidification and deamination caused by the proliferation and reproduction of microorganisms, resulting in protein decomposition and the formation of small molecular nitrogen-containing compounds, which subsequently release a certain amount of salt nitrogen substances. TVB-N serves as a crucial indicator for assessing meat product freshness [50]. As shown in Figure 5C, fried steaks initially exhibited a TVB-N value of 11.33 mg/100 g, which gradually increased with prolonged storage time. On the ninth day of storage, there was a significant rise in TVB-N content within the Butter group due to microbial colonization on beef surfaces [33]. By the 15th day of storage, the Butter group displayed a TVB-N value of 20.88 mg/100 g, indicating spoilage. RBW group had a TVB-N value of 16.92 mg/100 g; BW and CW groups showed values of 14.59 mg/100 g and 15.35 mg/100 g, respectively. Notably, oil gel frying demonstrated greater efficacy in inhibiting the increase in TVB-N content compared to other methods during equivalent storage periods. The TBARS value was utilized to quantify the extent of lipid oxidation, as depicted in Figure 5D. Initially, the TBARS value of fried steaks was recorded at 0.35 mg/kg, and throughout storage, the TBARS values for all steak groups exhibited an increasing trend. Notably, the Butter group consistently displayed significantly higher TBARS values compared to other groups during each storage period. By day 12, the TBARS value for the Butter group had reached 0.68 mg/kg, representing a notable increase of 0.33 mg/kg from its initial level. It is well-established that greater rancidity corresponds to poorer quality [33]. Generally speaking, when TBARS exceeds 0.5 mg/kg, it imparts a pronounced off-flavor [40,50]. After storage, the respective TBARS values for BW, CW, and RBW groups were measured at 0.53 mg/kg, 0.58 mg /kg, and 0.62 mg /kg, respectively. These three groups demonstrated varying degrees of rancidity with relatively slow growth rates and limited ranges during each time interval. It has been proven that gel frying technology leads to lower levels of fat oxidation compared to butter frying. The application of the oil-gel frying technique enables better control over steak quality changes by reducing storage losses, inhibiting TVB-N and TBARS growth, and thereby extending shelf life while enhancing overall quality.

## 4. Conclusions

In this study, Angus cattle naked eye steaks were selected to investigate the potential application of KPSO gel fried steaks. The findings demonstrated that different wax based formulations of KPSO gels significantly influenced quality factors (color, texture, cooking loss), volatile flavor compound production, and storage stability of fried steak. As the doneness and heating time increased for Butter steaks, the cooking loss rate was higher compared to gel oil steaks, resulting in a lower yield rate. Sensory composite scores indicated that medium well BW steaks received the highest rating. Therefore, consumers are recommended to choose gel oil steak when frying their steak to medium well or well done as it offers greater nutritional value at these stages. Further analysis detected a total of 69 volatile flavor compounds in the steak samples. PCA and cluster analysis based on flavor compounds revealed similar comprehensive scores between BW gel oil and Butter steak samples. During storage, the Butter group exhibited poorer performance with higher rates of storage loss and significant increases in TVB-N values and TBARS values; however, the gel group showed better results in these aspects. These findings demonstrate that oil-gel frying technology not only allows for better control over sensory quality but also reduces storage losses while inhibiting growth in TVB-N value and TBARS value levels thereby extending shelf life and improving overall steak quality. In conclusion, this study analyzed the effects of KPSO gel-fried steak on volatile flavor compounds and sensory quality providing valuable insights for quality control measures as well as future research endeavors related to fried steak.

## Figures and Tables

**Table 1:** Changes in color of different waxy based KPSO gels with Butter steak.

Degree of Sophistication		Rare	Medium Rare	Medium	Medium Well	Well Done
L*	BW	31.77 ± 0.48 ^c^	32.71 ± 0.47 ^b^	32.58 ± 0.06 ^bc^	34.26 ± 0.63 ^a^	35.04 ± 0.64 ^a^
	RBW	32.29 ± 0.23 ^c^	32.83 ± 0.25 ^bc^	33.45 ± 0.28 ^a^	33.67 ± 0.45 ^a^	33.38 ± 0.31 ^ab^
	CW	30.67 ± 0.21 ^d^	31.46 ± 0.32 ^c^	31.69 ± 0.26 ^bc^	31.99 ± 0.22 ^ab^	32.16 ± 0.09 ^a^
	Butter	32.38 ± 0.32 ^c^	32.99 ± 0.59 ^c^	34.32 ± 0.34 ^b^	35.37 ± 0.22 ^a^	35.67 ± 0.22 ^a^
a*	BW	7.50 ± 0.17 ^c^	7.85 ± 0.19 ^b^	8.17 ± 0.05 ^a^	8.45 ± 0.22 ^a^	7.63 ± 0.10 ^bc^
	RBW	7.70 ± 0.34 ^c^	9.55 ± 0.31 ^a^	8.92 ± 0.30 ^b^	9.53 ± 0.20 ^a^	7.62 ± 0.35 ^c^
	CW	8.29 ± 0.17 ^d^	9.34 ± 0.35 ^c^	10.40 ± 0.22 ^a^	9.77 ± 0.11 ^b^	9.76 ± 0.08 ^b^
	Butter	7.43 ± 0.21 ^c^	8.32 ± 0.35 ^b^	8.74 ± 0.16 ^ab^	8.82 ± 0.13 ^a^	7.54 ± 0.37 ^c^
b*	BW	10.90 ± 0.40 ^d^	11.91 ± 0.25 ^c^	13.09 ± 0.15 ^b^	13.45 ± 0.34 ^b^	14.21 ± 0.29 ^a^
	RBW	12.83 ± 0.63 ^d^	12.83 ± 0.30 ^d^	13.85 ± 0.20 ^c^	14.52 ± 0.15 ^b^	15.75 ± 0.46 ^a^
	CW	10.62 ± 0.31 ^c^	11.74 ± 0.25 ^b^	12.03 ± 0.55 ^bc^	11.94 ± 0.12 ^b^	12.54 ± 0.29 ^a^
	Butter	12.81 ± 0.26 ^b^	11.79 ± 0.13 ^c^	12.55 ± 0.24 ^b^	13.62 ± 0.34 ^a^	13.84 ± 0.25 ^a^

Note: L* represents Luminosity, a* represents the range from red to green, and b* represents the range from blue to yellow. Values with different lowercase letters in the same column are significantly different (*p* < 0.05).

**Table 2:** Sensory scores of KPSO gel versus Butter steak.

Sensory Attributes	Oil Sample	Rare	Medium Rare	Medium	Medium Well	Well Done
Color	BW	6.98 ± 0.33 ^a^	6.57 ± 0.35 ^a^	7.56 ± 0.13 ^a^	8.73 ± 0.28 ^a^	7.76 ± 0.25 ^a^
	RBW	6.24 ± 0.46 ^b^	6.54 ± 0.19 ^a^	7.51 ± 0.37 ^a^	8.49 ± 0.29 ^a^	7.59 ± 0.37 ^a^
	CW	6.28 ± 0.17 ^b^	6.11 ± 0.16 ^b^	7.62 ± 0.34 ^a^	8.24 ± 0.24 ^ab^	7.67 ± 0.26 ^a^
	Butter	6.34 ± 0.34 ^b^	6.62 ± 0.26 ^a^	7.39 ± 0.11 ^b^	8.12 ± 0.18 ^b^	7.12 ± 0.37 ^b^
Odor	BW	6.57 ± 0.27 ^b^	7.72 ± 0.35 ^a^	8.58 ± 0.38 ^a^	8.23 ± 0.13 ^b^	8.68 ± 0.26 ^a^
	RBW	7.55 ± 0.23 ^a^	7.73 ± 0.16 ^a^	8.63 ± 0.33 ^a^	8.37 ± 0.16 ^ab^	8.12 ± 0.19 ^b^
	CW	6.66 ± 0.14 ^b^	6.43 ± 0.32 ^c^	8.55 ± 0.32 ^a^	8.38 ± 0.27 ^ab^	8.31 ± 0.22 ^ab^
	Butter	7.78 ± 0.12 ^a^	7.27 ± 0.28 ^b^	8.14 ± 0.36 ^b^	8.45 ± 0.23 ^a^	8.52 ± 0.26 ^a^
Flavor	BW	6.34 ± 0.10 ^ab^	6.61 ± 0.07 ^b^	7.30 ± 0.02 ^c^	8.37 ± 0.07 ^b^	7.62 ± 0.04 ^a^
	RBW	6.21 ± 0.07 ^b^	6.85 ± 0.08 ^a^	7.46 ± 0.06 ^b^	8.28 ± 0.04 ^b^	7.08 ± 0.05 ^b^
	CW	6.40 ± 0.08 ^a^	6.38 ± 0.05 ^c^	7.35 ± 0.03 ^c^	7.75 ± 0.10 ^c^	7.17 ± 0.05 ^b^
	Butter	6.34 ± 0.10 ^ab^	6.51 ± 0.08 ^b^	7.67 ± 0.07 ^a^	8.58 ± 0.05 ^a^	6.43 ± 0.09 ^c^
Tenderness	BW	6.20 ± 0.05 ^d^	6.26 ± 0.02 ^b^	6.89 ± 0.08 ^c^	8.45 ± 0.18 ^a^	7.54 ± 0.04 ^b^
	RBW	6.26 ± 0.23 ^b^	6.25 ± 0.10 ^c^	7.13 ± 0.08 ^b^	8.22 ± 0.06 ^b^	7.64 ± 0.09 ^a^
	CW	6.23 ± 0.09 ^c^	6.30 ± 0.04 ^a^	7.12 ± 0.06 ^b^	7.83 ± 0.04 ^b^	7.06 ± 0.03 ^c^
	Butter	6.29 ± 0.08 ^a^	6.22 ± 0.05 ^d^	7.41 ± 0.04 ^a^	8.22 ± 0.05 ^c^	6.48 ± 0.05 ^d^
Juiciness	BW	6.34 ± 0.08 ^a^	6.31 ± 0.03 ^b^	7.27 ± 0.05 ^ab^	8.60 ± 0.06 ^a^	7.39 ± 0.04 ^a^
	RBW	6.21 ± 0.10 ^a^	6.38 ± 0.04 ^b^	7.38 ± 0.06 ^a^	8.70 ± 0.02 ^a^	7.10 ± 0.07 ^b^
	CW	5.71 ± 0.23 ^b^	6.12 ± 0.06 ^c^	7.21 ± 0.05 ^b^	8.09 ± 0.05 ^c^	6.87 ± 0.05 ^c^
	Butter	5.70 ± 0.32 ^b^	6.69 ± 0.04 ^a^	7.17 ± 0.10 ^b^	8.35 ± 0.11 ^b^	6.29 ± 0.03 ^d^
Chewiness	BW	6.48 ± 0.15 ^ab^	6.95 ± 0.29 ^a^	7.27 ± 0.31 ^ab^	8.03 ± 0.22 ^c^	8.03 ± 0.39 ^a^
	RBW	6.65 ± 0.25 ^a^	6.88 ± 0.32 ^a^	7.58 ± 0.23 ^a^	8.29 ± 0.17 ^a^	8.37 ± 0.19 ^a^
	CW	6.17 ± 0.18 ^c^	6.51 ± 0.11 ^b^	7.24 ± 0.11 ^b^	8.48 ± 0.16 ^a^	8.06 ± 0.33 ^a^
	Butter	6.34 ± 0.13 ^b^	6.38 ± 0.24 ^b^	7.37 ± 0.32 ^ab^	8.31 ± 0.22 ^a^	7.63 ± 0.21 ^b^
Overall Impression	BW	6.67 ± 0.43 ^b^	6.56 ± 0.16 ^b^	7.28 ± 0.36 ^c^	8.47 ± 0.31 ^a^	8.22 ± 0.24 ^ab^
	RBW	6.15 ± 0.48 ^c^	6.32 ± 0.25 ^bc^	7.92 ± 0.25 ^a^	8.76 ± 0.28 ^a^	8.34 ± 0.17 ^a^
	CW	6.73 ± 0.53 ^ab^	6.21 ± 0.18 ^c^	7.59 ± 0.16 ^b^	8.25 ± 0.14 ^b^	8.51 ± 0.37 ^a^
	Butter	6.83 ± 0.13 ^a^	6.96 ± 0.27 ^a^	7.04 ± 0.15 ^d^	8.38 ± 0.23 ^ab^	7.69 ± 0.43 ^b^

Note: Values with different lowercase letters in the same column are significantly different (*p* < 0.05).

## Data Availability

The original contributions presented in the study are included in the article, further inquiries can be directed to the corresponding authors.

## Abbreviations

Korean pine seed oil	KPSO
Beeswax	BW
Rice bran wax	RBW
Carnauba wax	CW
